# A Rare Case of Neuroendocrine Tumor Presenting as Isolated Scalp Swelling With Paroxysmal Symptoms

**DOI:** 10.7759/cureus.88202

**Published:** 2025-07-17

**Authors:** Vivek Kumar Dubey, Kush Pathak, Kavita Sijwali

**Affiliations:** 1 General Surgery, Ganesh Shankar Vidyarthi Memorial Medical College, Kanpur, IND; 2 Surgical Oncology, Ganesh Shankar Vidyarthi Memorial Medical College, Kanpur, IND

**Keywords:** gallium dotatate pet scan, neuroendocrine tumor, rare scalp tumor, scalp swelling, surgical oncology

## Abstract

We report a 36-year-old male with a nine-year history of recurrent left frontal scalp swelling following minor trauma, associated with local warmth, sweating, and a syncopal episode during fine-needle aspiration cytology (FNAC). Histopathology confirmed grade 2 neuroendocrine tumor (NET) with a Ki-67 index of 8-10%. 68Ga-DOTANOC positron emission tomography and computed tomography (PET-CT) showed no evidence of residual, recurrent, or primary somatostatin receptor-expressing disease, confirming this as a rare case of isolated scalp NET without an identifiable primary source. This is, to our knowledge, the first reported case of its kind. The patient’s syncope post-FNAC highlights the risk of carcinoid-like crisis from lesion manipulation. This case underscores the importance of a high index of suspicion, thorough immunohistochemical workup, and consideration of peri-procedural octreotide prophylaxis.

## Introduction

Neuroendocrine tumors may present with symptoms resembling carcinoid syndrome or crisis, particularly during interventions such as biopsies or surgeries. Peri-procedural management with octreotide has been advocated in symptomatic cases to mitigate risk [1,2]. Neuroendocrine tumors (NETs) are uncommon neoplasms, constituting less than 2% of all malignancies [3]. They primarily arise from the gastrointestinal tract or bronchopulmonary system. Metastatic NETs involving the skin are extremely rare (<1%), and scalp involvement is even more unusual [4,5]. NETs commonly manifest with hormonal syndromes or as incidental findings. In this case, a long-standing post-traumatic scalp lesion eventually revealed a NET, complicated by a syncopal episode during fine-needle aspiration cytology (FNAC). This case is unique due to the absence of any detectable primary tumor on 68Ga-DOTANOC positron emission tomography and computed tomography (PET-CT), indicating a solitary cutaneous NET of unclear origin, which has not been previously documented.

## Case presentation

Clinical history

**Figure 1:**
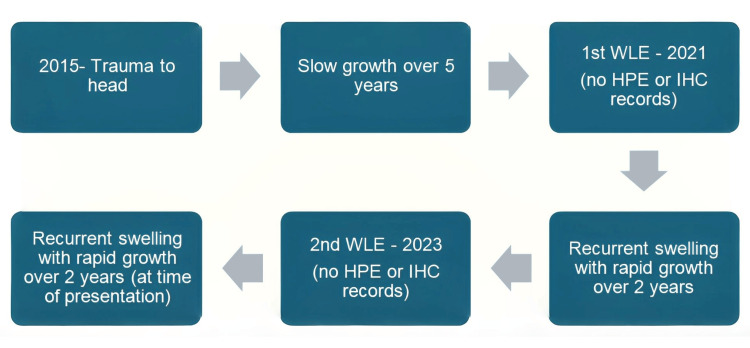
Timeline of lesion progression and interventions. After trauma to head in 2015, the swelling grew over five years, leading to wide local excision in 2021, recurred over two years, followed by wide local excision again in 2023. After the second recurrence, the patient presented to our side. WLE: wide local excision; HPE: histopathological examination; IHC: immunohistochemistry.

A 36-year-old male presented with recurrent swelling over the left frontal scalp. It originated following blunt trauma in 2016, evolving from a painless 1 cm nodule to a progressively enlarging mass over five years. He underwent excisions in 2021 and 2023, but both resulted in recurrence within two years, respectively. No histopathological examination was performed during prior surgeries. The complex appearance, management, recurrences, and final presentation to our side can be summarized in a flowchart diagram as shown in Figure 1.

Examination and imaging

**Figure 2:**
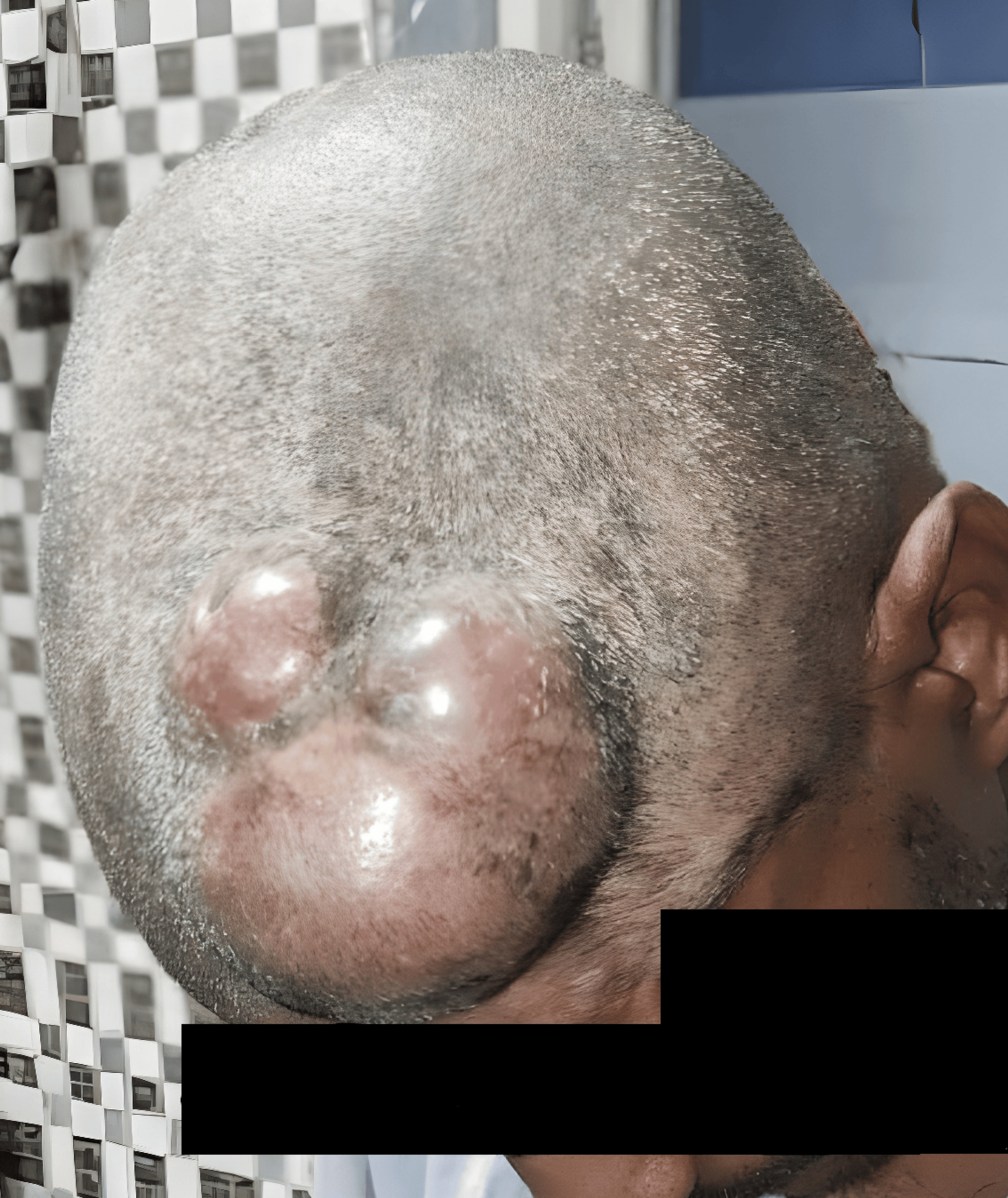
Preoperative image of the lesion showing a warm, soft, localized, slightly tender, bosselated, non-ulcerated swelling in the left frontal region of the scalp with fixity to skin and free base.

On examination, a 6 cm × 5 cm, warm on touch, soft, localized, slightly tender, bosselated, non-ulcerated swelling was noted. The lesion was fixed to the skin but non-adherent to the base of the skull, with no regional lymphadenopathy palpable, as shown in Figure 2. 

Contrast-enhanced MRI showed a 6.2 cm × 5.8 cm well-defined subgaleal lesion with no bony or intracranial involvement and no regional lymph node deposits.

Fine-needle aspiration cytology (FNAC) and paroxysmal symptoms

In 2025, FNAC suggested a small round cell tumor (likely non-Hodgkin’s lymphoma). Immediately after the procedure, the patient experienced a syncopal episode with hyperthermia and profuse sweating, resolving spontaneously in 30 minutes. Such neuroendocrine crisis-like episodes are rarely reported but can occur due to serotonin or neuropeptide release [6-8].

Surgical management

**Figure 3:**
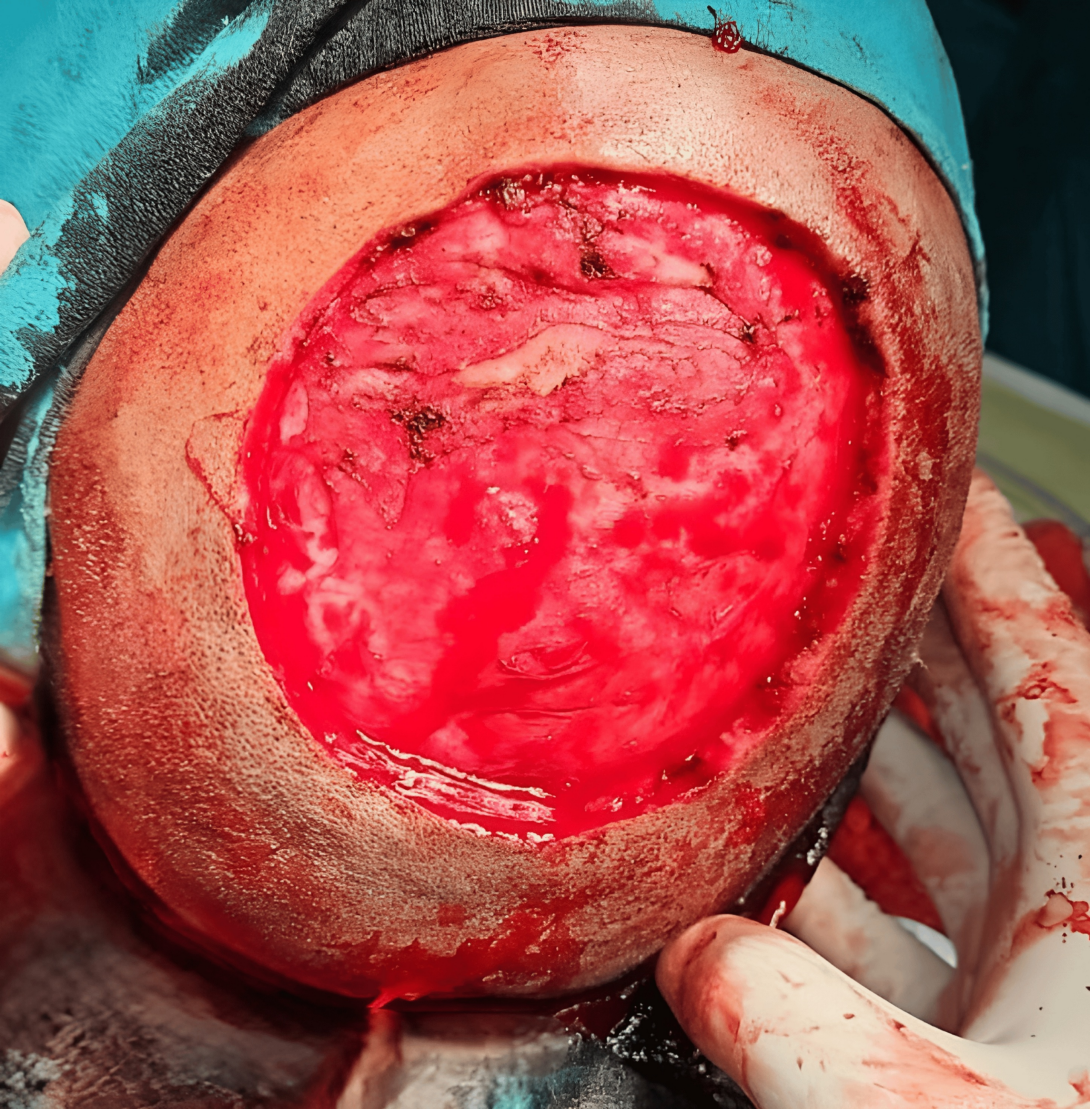
Intraoperative image showing large post-excisional defect. After carefully achieving hemostasis, the wound bed was ready to be covered with a rotational scalp flap.

Wide local excision was performed with 1 cm margins. The tumor was adherent to the galea but easily separable from the periosteum. Bleeding scalp margins were secured by diathermy, and hemostasis was achieved. Once all active bleeding points were secured as shown in Figure 3, flap cover and reconstruction was initiated.

**Figure 4:**
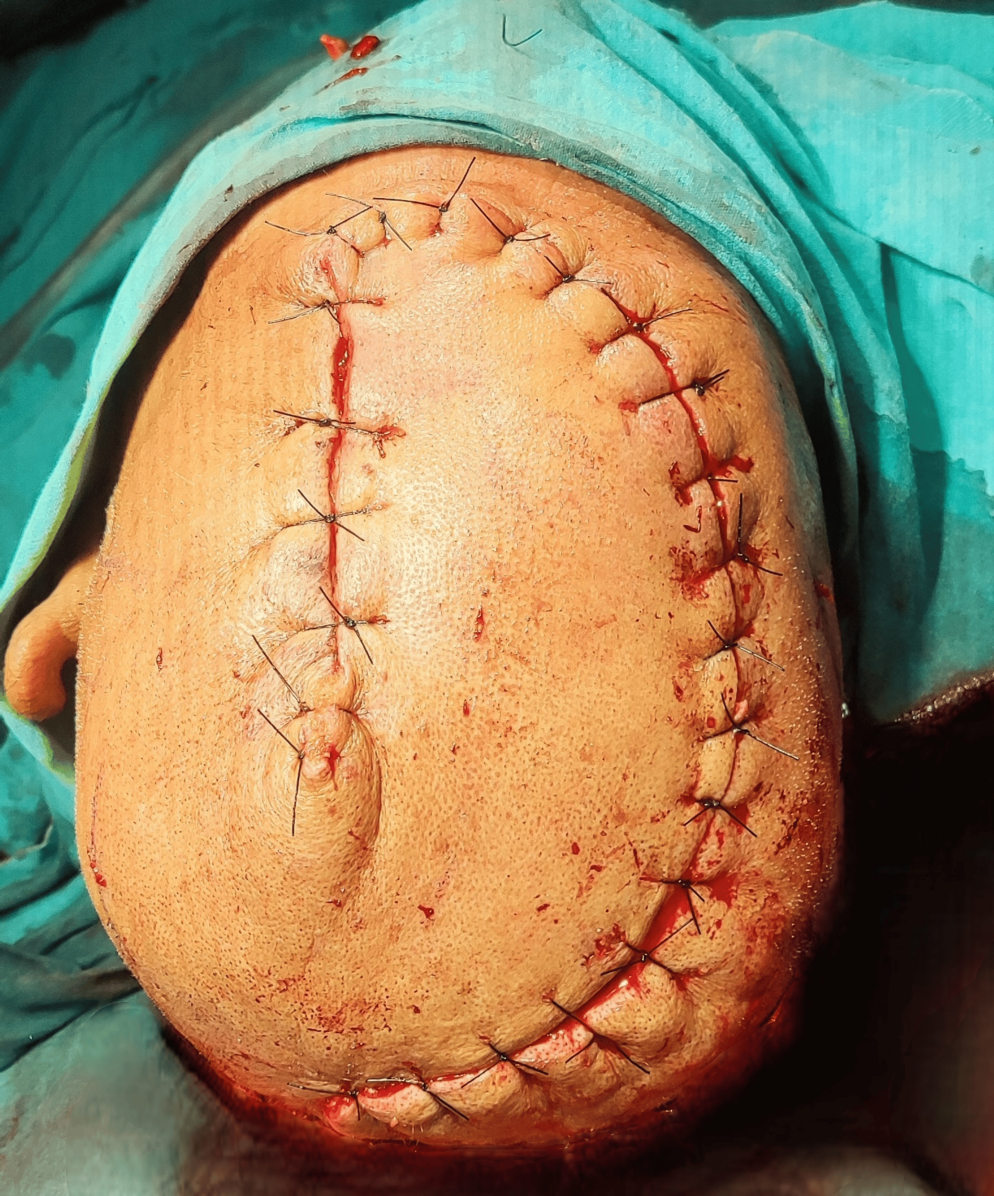
Large defect was covered using a scalp rotational flap. Non-absorbable sutures were secured in an interrupted fashion.

The large post-excisional defect was further covered with a rotational flap of the scalp. Flap was raised and carefully rotated to cover the large defect, securing it with non-absorbable interrupted sutures, as shown in Figure 4. Regular aseptic cleaning and dressing were done to keep the wound free of infection and promote healing.

Histopathology and immunohistochemistry

Histopathology and immunohistochemistry microscopy revealed dermal and subcutaneous infiltration by uniform tumor cells with “salt-and-pepper” chromatin and low mitotic activity. Immunohistochemistry (IHC) was strongly positive for chromogranin, synaptophysin, and focally for pancytokeratin (Pan-CK); negative for cluster of differentiation 45 (CD45), cytokeratin 7 (CK7), cytokeratin 20 (CK20), S100, and Melan-A (also known as melanoma antigen recognized by T-cells or MART-1). Ki-67 index was 8-10%, consistent with a grade 2 NET [9]. Margins were clear (R0 resection). A comparison between the commonly associated tumor markers in NET and their differentials is shown in Table 1.

**Table 1:** IHC profile comparison table. NET: neuroendocrine tumor; IHC: immunohistochemistry; CK20: cytokeratin 20.

Feature	This case (NET)	Merkel cell carcinoma	Lymphoma
Synaptophysin	Positive	Rarely positive	Negative
Chromogranin	Positive	Negative	Negative
CK20	Negative	Positive (80%)	Negative
Ki-67 Index	8–10%	Usually >20%	Variable

Postoperative imaging

A whole-body 68Ga-DOTANOC PET-CT performed six weeks postoperatively revealed no abnormal somatostatin receptor-expressing lesions in the scalp, chest, abdomen, or bones. Mild mucosal thickening was noted in the bilateral ethmoid and maxillary sinuses, suggestive of an inflammatory process. A few DOTANOC-avid mediastinal lymph nodes were seen, but were non-significant. No focal abnormal DOTANOC uptake was detected elsewhere, confirming the absence of residual, recurrent, or metastatic disease.

## Discussion

Rarity and diagnostic challenge

Cutaneous and scalp NETs are exceedingly rare. Wang et al. reported only isolated cases of scalp metastases from gastrointestinal NETs [5]. Primary cutaneous NETs remain controversial, and often extensive workup fails to identify the source in 10-15% of cases [3]. This case meets that rare category, especially with a negative DOTANOC PET-CT.

Syncope and crisis-like event

FNAC-induced syncopal and carcinoid-like symptoms are rarely documented. Neurohumoral release from manipulation may mimic carcinoid crisis [6,8,10] or induce reflex syncope via carotid sinus stimulation [7]. The autonomic symptoms in this case suggest neuropeptide-mediated vasodilation, similar to carcinoid syndrome seen in gastrointestinal NETs [11].

Treatment and prognosis

Wide excision remains the treatment of choice. Long-term surveillance is critical. Peri-procedural octreotide prophylaxis should be considered in cases with symptomatic NETs to prevent neuroendocrine crisis [1,2,12]. Regular imaging and clinical follow-up are essential due to the risk of recurrence or late identification of a primary focus.

## Conclusions

This is the first reported case of a grade 2 NET localized solely to the scalp, with no identifiable primary or systemic disease, confirmed by DOTANOC PET-CT. The unusual history of trauma and syncope during FNAC adds clinical intrigue. This underscores the need for high diagnostic suspicion, careful IHC interpretation, and functional imaging to guide appropriate management and prevent peri-procedural crises. The patient has been kept on regular follow-up to detect any early symptoms, and repeat scans are planned after an interval of three months to uncover any hidden foci, which may have not been revealed in this scan.
